# Sexual Dimorphism in Digit Ratios Derived from Dorsal Digit Length among Adults and Children

**DOI:** 10.3389/fendo.2017.00041

**Published:** 2017-03-06

**Authors:** Sanjay Kumar, Martin Voracek, Maharaj Singh

**Affiliations:** ^1^Department of Psychology, D.A.V. College, Muzaffarnagar, India; ^2^Department of Basic Psychological Research and Research Methods, Faculty of Psychology, University of Vienna, Vienna, Austria; ^3^College of Professional Studies, Marquette University, Milwaukee, WI, USA; ^4^Aurora Research Institute, Milwaukee, WI, USA

**Keywords:** digit ratio (2D:4D), alternative digit ratios, flexion creases, dorsal finger length, sex differences, prenatal testosterone

## Abstract

Sexual dimorphism in ventrally measured digit ratios (2D:4D and other) has been related to prenatal sex-hormone levels. In the present series of three studies, we measured all digit lengths (excluding the thumb) on the dorsal, rather than the ventral, side of left and right hands and investigated the sexual dimorphism in digit ratios in three independent samples, two of them comprising adults (Study I, *N* = 104; Study II, *N* = 154), and one further, comprising kindergarten children (Study III, *N* = 64). Results show that men have lower digit-ratio values compared to women in digit ratios that include digit 5 as one of the constituents of the ratio (i.e., the 4D:5D, 3D:5D, and 2D:5D ratios). Boys have lower values compared to girls for the 4D:5D and 3D:5D ratios, and there is a similar trend of sexual dimorphism in the 2D:5D ratio. Thus, based on the evidence from dorsally measured digit ratios, the present findings from three samples are consistent with the idea that early sex-hormonal effects might be stronger for digit ratios involving digit 5, as compared to the classic, and frequently studied, ventrally measured 2D:4D ratio.

## Introduction

Research suggests that sexual dimorphism in the second-to-fourth digit ratio (2D:4D), when finger length is measured on the ventral side (i.e., the length between the fingertip and the proximal basal crease), is related with prenatal sex-hormone levels (1, 2). Individuals affected with conditions entailing high prenatal testosterone levels, such as congenital adrenal hyperplasia (3, 4), autism (5), or women with male co-twins (6), present lower 2D:4D ratios, measured ventrally, as compared with controls. Further, sex-chromosomal disorders, such as complete androgen insensitivity syndrome (7) and Klinefelter syndrome (8), support female-typical 2D:4D ratios in genetic males. Thus, the 2D:4D ratio, measured ventrally, might be related to prenatal sex-hormone levels. However, theoretically, digit ratios based on bone-length measurement, should be even more closely related to the prenatal sex-hormonal milieu, because it is the bone which determines most of the length of a finger, and sex hormones affect the posterior Hox genes, which are an important determinant of the relative growth of bones in general (9). This view is supported by studies reporting sexual dimorphism in the 2D:4D ratio based on bone length (10–13). However, probably because of the properties of non-hazardousness and easy measurement, digit ratios derived from ventral digit length have preferably been used in research.

Studies comprising several (i.e., alternative) digit ratios derived from ventral digit length have reported stronger sexual dimorphism occurring in digit ratios having digit 2 as one of the constituents of the ratio (14–17). On the other hand, studies comprising several digit ratios derived from metacarpal-bone length have reported stronger sexual dimorphism occurring in digit ratios having digit 5 as one of the constituents of the ratio [in gorillas (18); in humans (19)]. In addition, studies have also reported a pattern of similar sexual dimorphism in digit ratios (2D:4D) derived from metacarpal and phalangeal bone lengths (12). Thus, the pattern of sexual dimorphism in digit ratios derived from phalangeal bone length likely is different from the pattern of sexual dimorphism in the corresponding digit ratios, when these are derived from ventral digit-length measurements. Because sex differences in digit ratios from phalangeal bone length are noticeably stronger than those in the corresponding metacarpal-bone digit ratios [four times stronger with regard to the 2D:4D ratio (12)] and because the effect size for sex differences in metacarpal-bone digit ratios is close to *d* = 0.5 [for ratios with digit 5 as one of the digits (19)], a larger sexual dimorphism is expected for digit ratios from phalangeal bone lengths. Thus, dissimilar to prior studies which reported effects of small-to-medium-size [i.e., *d* = 0.2–0.5 (20)], the study of digit ratios derived from phalangeal bone length is expected to yield larger sex differences.

Prior related research has not attempted a study of the sexual dimorphism in all digit ratios derived from phalangeal bone length. One possible reason for this fact could be that common machines emit X-rays divergently from the source, and thus skewed images could appear for peripheral areas. Such X-ray images may contain systematic error when the variables of interest are the length ratios between length measures of focal and peripheral areas of the hand (for example, the ratio between digit 3 and digit 5 vs. the ratio between digit 3 and digit 4). Of note, McFadden and Bracht (18, 19) conducted both of their studies, involving the digit ratios of all metacarpal-bone lengths, on skeletons.

Similar to ventral finger-length measurement, dorsal finger-length measurement is easy, non-hazardous, and expected to be reliable (21). Dorsal digit length mainly includes fingertip fat and the length determined by the ray (i.e., chain) of the three (namely, proximal, intermediate, and distal) phalangeal bones. Studies have shown that size of fingertip fat is not sexually dimorphic and has no relationship with 2D:4D (10, 13, 22). As well, dorsal measurement of digit lengths does not include the finger flexion creases, which might be one factor of measurement error and unreliability. For these combined reasons, it is conceivable that, as compared to digit ratios derived from ventral digit length, digit ratios derived from dorsal digit length might more appropriately represent the actual sexual dimorphism effect in digit ratios derived from phalangeal bone length. In contrast to measuring phalangeal bone length, it is possible to measure accurately all dorsal digit lengths in a living hand. Hence, investigations of digit ratios derived from dorsal digit length may well be more suitable for a better understanding of the patterns of sexual dimorphism found in digit ratios.

Therefore, in the present series of studies, we measured dorsal digit length of all fingers (excluding the thumb) in both hands of study participants and compared the magnitude of the sexual dimorphism in the digit ratios calculated from these finger-length measurements. As elaborated on above, digit ratios derived from dorsal digit length may be suitable proxies of digit ratios derived from phalangeal bone length. The objective of the present research was therefore to understand the patterns of sexual dimorphism in digit ratios based on phalangeal bone length by studying dorsally measured digit ratios, which are expected to show patterns of sexual dimorphism similar to the one found in metacarpal-bone digit ratios (19). Accordingly, we hypothesized that stronger sexual dimorphism would occur in dorsally measured digit ratios involving digit 5 as one of the constituents of the ratio.

Studies have reported that sexual dimorphism in the 2D:4D ratio is established much earlier than puberty (2, 10, 23, 24). Similar to the case of the classic 2D:4D ratio, it is possible that sexual dimorphism in other (alternative) digit ratios also is determined early on. However, no prior research has focused on sexual dimorphism in alternative (other than 2D:4D) digit ratios among children [with the exception of the 3D:4D ratio (11)]. Moreover, studies also have shown that comparatively large age-related changes occur in digit ratios involving digit 5 (24). Thus, investigating sexual dimorphism in several digit ratios among adults as well as among children may be fruitful for a better understanding of these patterns and developmental changes therein.

We examined our hypotheses in two independent samples of adults (Studies I and II). This strategy of testing the same research hypotheses in more than just a single sample (i.e., not leaving replication of initial findings to other researchers and the future) constitutes an attempt of internal replication and is known as the discovery-replication sample approach. In addition, we examined the same research hypotheses in a further sample, comprised of children (Study III). All the three studies reported here received approval of the Ethics Committee of the D.A.V. College.

## Study I

### Method

#### Participants

A sample of 104 right-handed college students and teachers, ranging in age from 16 to 48 years (age M = 23.4, SD = 6.1 years; men: *n* = 51, women: *n* = 53), was selected from Muzaffarnagar city (Western Uttar Pradesh, India). Because left-handed vs. right-handed individuals may differ with regard to digit ratios (25), only right-handers were selected. Prior to measurement, participants were inquired about hand injuries and provided written informed consent to study participation.

#### Measurement of Digit Lengths and Ratios

**Figure 1:**
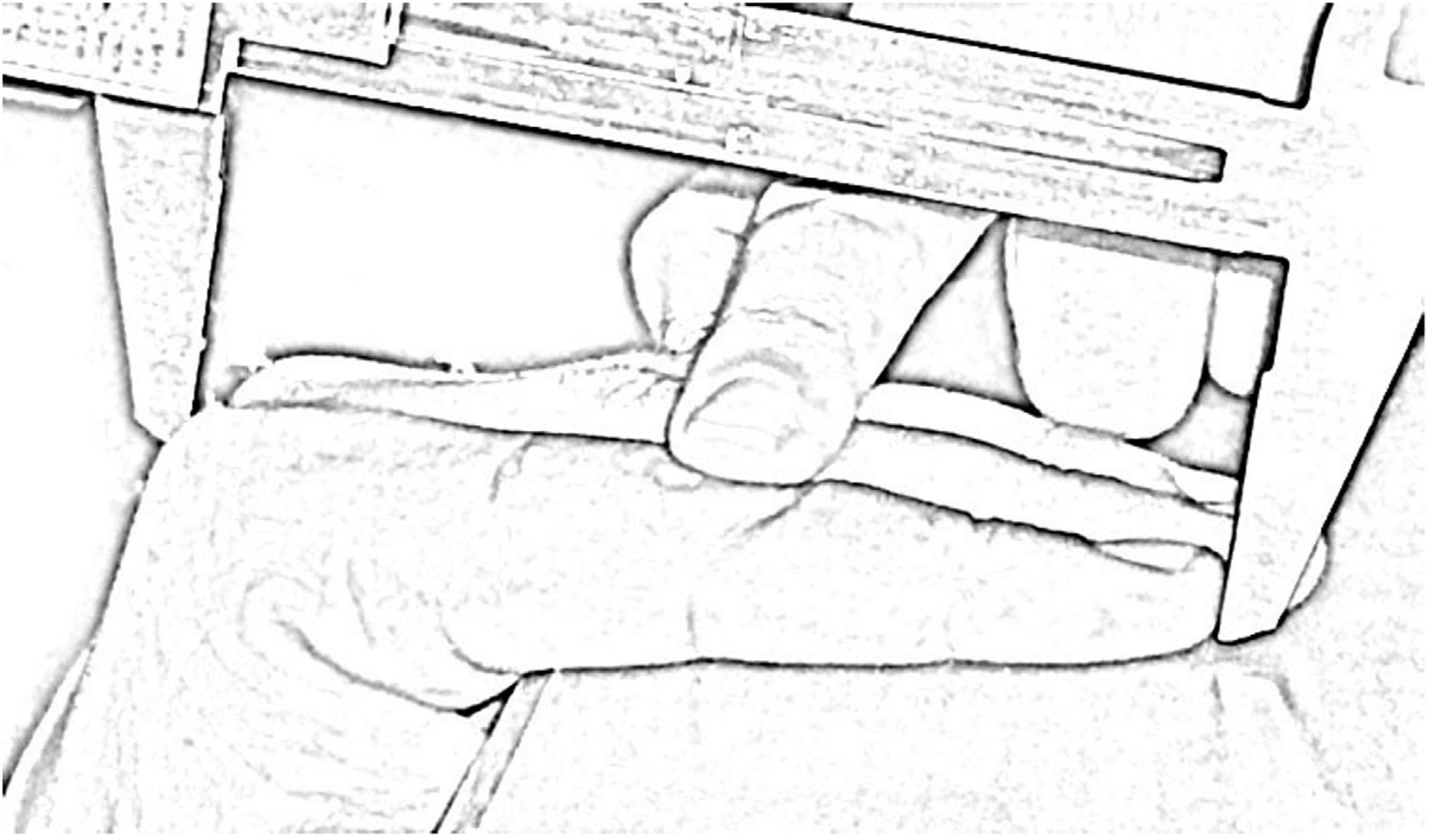
**Sketch depicting the measurement procedure of dorsal digit length using vernier calipers**. The finger is gently pressed at the phalangeal joint, in order to remain straight during the measurement.

Digit lengths were directly measured dorsally (as opposed to ventral measurements, as employed in past research), using vernier calipers with an accuracy level of 0.1 mm, by one researcher (Sanjay Kumar). Dorsal digit length includes the distance between the tip of finger and the dorsal base of the proximal phalanx. Measurements were taken by requesting participants to put their hand on the edge of a smooth table, so that their fingers make an angle of 90° to the palm (Figure 1). Per participant, eight measurements of four fingers were taken in both hands: 2D, 3D, 4D, and 5D lengths, both right and left. We calculated all possible digit ratios (six per hand) for both hands.

#### Reliability of Digit Measures

Repeated measurements of the dorsal length of the second to fifth digits in 40 hands (20 men and 20 women) were taken. Intraclass correlations between these measurements were high [all *p*s < 0.001; for mixed-effects model with absolute-agreement definition; see Ref. (26)]; 2D length: ICC = 0.997; 3D length: ICC = 0.997; 4D length: ICC = 0.997; 5D length: ICC = 0.995; 2D:3D ratio: ICC = 0.84; 2D:4D ratio: ICC = 0.96; 2D:5D ratio: ICC = 0.90; 3D:4D ratio: ICC = 0.90; 3D:5D ratio: ICC = 0.84; and 4D:5D ratio: ICC = 0.80. The magnitude of error between repeated measurements, construed as percentage of the size of the variable measured, known as the relative technical error of measurement (rTEM), likewise was small for digit lengths (2D length: 0.57%; 3D length: 0.60%; 4D length: 0.60%; and 5D length: 0.86%) and for digit ratios (2D:3D ratio: 0.88%; 2D:4D ratio: 0.67%; 2D:5D ratio: 1.1%; 3D:4D ratio: 0.73%; 3D:5D ratio: 1.06%; and 4D:5D ratio: 1.06%). Hence, the measurements of digit lengths and of digit ratios were highly repeatable.

#### Analysis

We calculated an analysis of variance (ANOVA) model with hand (right vs. left) and digit ratio (six digit ratios in a hand) as within-subject factors and participant sex (male vs. female) as the between-subjects factor. For ease of interpretation of results, we additionally calculated effect sizes (Cohen’s *d*), quantifying the magnitude of the sex differences in the digit ratios. Analysis for Studies II and III proceeded similarly.

### Results and Discussion

The repeated-measures ANOVA (after Greenhouse–Geisser correction) showed an effect of digit ratio, *F*(1.8, 180.9) = 6,175, *p* < 0.001, and a two-way interaction between sex and digit ratio, *F*(1.8, 180.9) = 10.7, *p* < 0.001. Participant sex as the between-subjects factor was significant as well, *F*(1, 102) = 7.8, *p* = 0.006.

Mean comparisons showed that women have higher 3D:5D, *F*(1, 102) = 10.4, *p* = 0.002; 4D:5D, *F*(1, 102) = 18.3, *p* < 0.001; and 2D:5D, *F*(1, 102) = 8.15, *p* = 0.005, ratios than men. There were no sex differences in the 2D:3D, *F*(1, 102) = 0.3, *p* = 0.6; 2D:4D, *F*(1, 102) = 0.9, *p* = 0.4; and 3D:4D, *F*(1, 102) = 0.6, *p* = 0.4, ratios (Table 1). Thus, the results of Study I support the hypothesis that a comparatively stronger sexual dimorphism occurs in digit ratios that include digit 5. The order of the effect size of the sexual dimorphism in the digit ratios was 4D:5D > 3D:5D > 2D:5D, and these sex differences were of medium to large size (*d* = −0.56 to −0.83). Table 1 also shows that the effect of higher digit ratios in women than in men occurred more clearly for left-hand than for right-hand digit ratios.

**Table 1:** **Descriptive statistics and sex differences in dorsally measured digit ratios for Study I sample (51 men, 53 women)**.

	Left hand					Right hand					Average hand				
	Men		Women			Men		Women			Men		Women		
	M	SD	M	SD	*d*	M	SD	M	SD	*d*	M	SD	M	SD	*d*
2D:3D	0.892	0.019	0.891	0.015	0.06	0.892	0.02	0.889	0.021	0.15	0.892	0.018	0.890	0.016	0.12
2D:4D	0.933	0.021	0.933	0.021	0.00	0.937	0.029	0.929	0.022	0.31	0.935	0.023	0.931	0.019	0.19
2D:5D	1.155	0.022	1.179	0.044	**−0.72**^a^	1.153	0.043	1.168	0.040	−0.36^d^	1.154	0.029	1.173	0.039	**−0.56**^b^
3D:4D	1.046	0.014	1.047	0.019	−0.06	1.051	0.020	1.046	0.016	0.28	1.048	0.014	1.046	0.015	0.14
3D:5D	1.296	0.028	1.323	0.050	**−0.69**^a^	1.293	0.046	1.314	0.041	**−0.48**^c^	1.295	0.032	1.319	0.043	**−0.64**^b^
4D:5D	1.239	0.024	1.264	0.036	**−0.83**^a^	1.231	0.044	1.257	0.033	**−0.68**^a^	1.235	0.029	1.260	0.032	**−0.82**^a^

*Values of |d| > 0.40 are shown in bold*.

*^a^p < 0.001*.

*^b^p < 0.01*.

*^c^p < 0.05*.

*^d^p < 0.10*.

## Study II

### Method

#### Participants

A new sample of 154 right-handed college students, aged between 17 and 28 years (age M = 19, SD = 1.9 years; men: *n* = 86, women: *n* = 68), was selected from Muzaffarnagar city. Study participants provided written informed consent to participate.

#### Measurement of Digit Lengths and Ratios

Similar to Study I, eight finger-length measurements on the dorsal side of both hands were taken and all possible digit ratios (six per hand) derived there from. Digit lengths were measured directly, using vernier calipers measuring with 0.1 mm accuracy level. A paid research assistant, blind to the research hypotheses, did the measurements. Hence, these measurement and the digit ratios calculated there from were independent from those of Study I (for which author Sanjay Kumar did the measurements).

### Results and Discussion

The repeated-measure ANOVA (after Greenhouse–Geisser correction) showed an effect of digit ratio, *F*(2, 306.6) = 10,277, *p* < 0.001, and a two-way interaction between participant sex and digit ratio, *F*(2, 306.6) = 5.81, *p* = 0.003. The between-subjects factor participant sex was significant as well, *F*(1, 152) = 8.25, *p* = 0.005.

Mean comparisons showed that women have higher 4D:5D, *F*(1, 152) = 9.8, *p* = 0.002; 3D:5D, *F*(1, 152) = 8.1, *p* = 0.005; and 2D:5D, *F*(1, 152) = 8.6, *p* = 0.004, ratios than men. There was no difference in 2D:3D, *F*(1, 152) = 0.1, *p* = 0.7; 2D:4D, *F*(1, 152) = 0.2, *p* = 0.6; and 3D:4D, *F*(1, 152) = 0.1, *p* = 0.8, ratios (Table 2). Thus, the results of Study II replicated the main findings of Study I and supported the hypothesized stronger sexual dimorphism in digit ratios involving digit 5. The order of the strength of the sex differences in the digit ratios was 4D:5D > 2D:5D ≥ 3D:5D, and effects were of medium size (*d* = −0.48 to −0.51). Interestingly, similar to Study I, the sex differences observed in the digit ratios again were stronger for the left hand than for the right hand (see Table 2).

**Table 2:** **Descriptive statistics and sex differences in dorsally measured digit ratios for Study II sample (86 men, 68 women)**.

	Left hand					Right hand					Average hand				
	Men		Women			Men		Women			Men		Women		
	M	SD	M	SD	*d*	M	SD	M	SD	*d*	M	SD	M	SD	*d*
2D:3D	0.895	0.016	0.894	0.016	0.06	0.893	0.017	0.896	0.016	−0.18	0.894	0.015	0.895	0.014	−0.06
2D:4D	0.937	0.019	0.937	0.022	0.00	0.935	0.021	0.938	0.020	−0.15	0.936	0.019	0.937	0.019	−0.08
2D:5D	1.169	0.034	1.184	0.033	**−0.45**^b^	1.164	0.043	1.180	0.033	**−0.42**^b^	1.166	0.036	1.182	0.030	**−0.48**^b^
3D:4D	1.047	0.019	1.047	0.017	0.00	1.047	0.020	1.048	0.019	−0.05	1.047	0.017	1.048	0.015	−0.04
3D:5D	1.306	0.040	1.324	0.031	**−0.50**^b^	1.303	0.042	1.318	0.040	−0.37^c^	1.304	0.039	1.321	0.032	**−0.47**^b^
4D:5D	1.248	0.029	1.264	0.027	**−0.57**^a^	1.244	0.041	1.258	0.031	−0.38^c^	1.246	0.033	1.261	0.025	**−0.51**^b^

*Values of |d| > 0.40 are shown in bold*.

*^a^p < 0.001*.

*^b^p < 0.01*.

*^c^p < 0.05*.

## Study III

### Method

#### Participants

A sample of 64 right-handed kindergarten children, aged between 3 and 7.6 years (age M = 5.04, SD = 1.22 years; age distribution: 3–4 years = 34.4%, 4–5 years = 21.9%, 5–6 years = 17.2%, 6–7 years = 25%, >7 years = 1.6%; boys: *n* = 31, girls: *n* = 33), was selected from a school in Muzaffarnagar city. Children’s parents provided written informed approval for their children participating in the study.

#### Measurement of Digit Lengths and Ratios

Similar to the adult samples of Studies I and II, eight measurements of four fingers on the dorsal side of both hands were taken. A female research assistant, blind to the hypothesis, used vernier calipers measuring to 0.1 mm accuracy level to measure digit lengths directly. From these finger-length measurements, all possible digit ratios (six per hand) were calculated.

#### Reliability of Digit Measures

Repeated measurements of the second and fourth digits on the dorsal side of the right hand of 36 children (age M = 5.6, SD = 0.81 years) were taken. High intraclass correlations (*p*s < 0.001; mixed-effects model with absolute-agreement definition) and low rTEMs were found for digit lengths and digit ratios (length of 2D: ICC = 0.98, rTEM = 1.09%; length of 4D: ICC = 0.99, rTEM = 0.84%; and 2D:4D ratio: ICC = 0.83, rTEM = 1.54%). This shows that the measurements of digit lengths and digit ratios were also highly repeatable for the children sample of Study III.

### Results and Discussion

Prior studies have shown that age-related changes occur in digit ratios among children (11). However, because no significant age effect for the Study III data was observed, all results are reported without entering age as a covariate in the ANOVA model.

The repeated-measures ANOVA (after Greenhouse–Geisser correction) showed an effect of digit ratio, *F*(1.7, 103) = 2,332, *p* < 0.001, and a two-way interaction between participant sex and digit ratio, *F*(1.7, 103) = 5.5, *p* = 0.008. The between-subjects factor (participant sex) was also significant, *F*(1, 62) = 4.3, *p* = 0.04.

Mean comparisons showed that the sex effect (girls having higher digit ratio than boys) was significant for the 3D:5D, *F*(1, 62) = 7.2, *p* = 0.009 and 4D:5D, *F*(1, 62) = 6.1, *p* = 0.017, ratios, was nominally not significant for the 2D:5D ratio, *F*(1, 62) = 3.4, *p* = 0.07, and also not significant for the 2D:3D, *F*(1, 62) = 2.4, *p* = 0.13; 2D:4D, *F*(1, 62) = 0.4, *p* = 0.5; and 3D:4D, *F*(1, 62) = 1.1, *p* = 0.3, ratios (Table 3). Thus, the results of Study III support the hypothesis that stronger sexual dimorphism occurs in digit ratios with digit 5 as one of the constituents of the ratio. The order of effect sizes for the sex differences was 3D:5D > 4D:5D > 2D:5D, and these effects were of medium size (*d* = −0.46 to −0.68).

**Table 3:** **Descriptive statistics and sex differences in dorsally measured digit ratios for Study III sample (31 boys, 33 girls)**.

	Left hand					Right hand					Average hand				
	Boys		Girls			Boys		Girls			Boys		Girls		
	M	SD	M	SD	*d*	M	SD	M	SD	*d*	M	SD	M	SD	*d*
2D:3D	0.900	0.020	0.893	0.022	0.33	0.906	0.021	0.899	0.021	0.33	0.903	0.017	0.896	0.019	0.39
2D:4D	0.943	0.029	0.941	0.026	0.07	0.953	0.022	0.949	0.027	0.16	0.948	0.023	0.945	0.022	0.13
2D:5D	1.167	0.060	1.195	0.046	**−0.53**^b^	1.182	0.038	1.194	0.058	−0.25	1.175	0.040	1.195	0.046	**−0.46**^c^
3D:4D	1.048	0.020	1.054	0.021	−0.29	1.052	0.024	1.055	0.023	−0.13	1.050	0.018	1.055	0.016	−0.29
3D:5D	1.297	0.066	1.339	0.054	**−0.70**^a^	1.305	0.041	1.328	0.063	**−0.44**^c^	1.301	0.044	1.334	0.053	**−0.68**^a^
4D:5D	1.238	0.064	1.271	0.050	**−0.58**^b^	1.240	0.032	1.259	0.051	**−0.46**^c^	1.239	0.037	1.265	0.045	**−0.63**^b^

*Values of |d| > 0.40 are shown in bold*.

*^a^p < 0.01*.

*^b^p < 0.05*.

*^c^p < 0.10*.

It is observable from Table 3 that the effect of higher digit ratios in girls than in boys occurred more strongly for the left hand than for the right hand. Thus, the pattern of sex differences in digit ratios among children in Study III was similar to that found among adults in the Studies I and II data.

## General Discussion

The present three studies report a pattern of sexual dimorphism from dorsally measured digit ratios similar to the pattern of sexual dimorphism reported by earlier studies for digit ratios derived from metacarpal-bone length (19). Because digit ratios derived from dorsal digit length might suitably reflect digit ratios derived from phalangeal bone length, the present evidence supports and extends the findings of Robertson et al. (12). Thus, it is conceivable that a stronger sexual dimorphism occurs in digit ratios that have digit 5 as one of the constituents in the ratios, as derived from phalangeal bone length.

Previous studies have reported patterns of noticeable ethnic and geographic differences in the 2D:4D ratio, either measured ventrally (27, 28) or from bone lengths (11). For both ventral and bone digit ratios, Europeans populations (ventral measurements: 0.985–0.994; bone measurements: 0.905–0.913) present higher 2D:4D, followed by Asian populations (ventral: 0.983–0.989; bone: 0.902–0.914), and African populations (ventral: 0.977–0.986; bone: 0.891–0.90) (11, 28). For dorsal length measurements, 2D:4D data are available for European populations [men: 0.96 (21)], which are higher than those reported for the samples from India in the present studies (0.94). Thus, the pattern of ethnic and geographic differences in 2D:4D derived from either dorsal, ventral, or bone digit length appears to be similar. The present study reports a pattern of sexual dimorphism in digit ratios similar to the one reported by McFadden and Bracht (19) for a Caucasian population. Thus, the occurrence of a relatively longer digit 5 in men than in women does not seem to depend on ethnicity or geographic region. Indeed, research suggests that, for the 2D:4D ratio, sex differences generalize across ethnic groups and world regions (11, 27, 28). It would be interesting to study the pattern of sexual dimorphism in dorsally measured digit ratios among African, European, and other populations.

The pattern of sexual dimorphism in dorsal digit ratios, as reported here, differs from the one that has been found for ventrally measured digit ratios (14–17). Because the main difference between dorsal and ventral digit lengths is the position of the (ventrally located) flexion creases, it appears conceivable that these could be the factor determining the differences between the patterns of sexual dimorphism in digit ratios derived from dorsal vs. ventral digit lengths. Furthermore, the tendency of a stronger sexual dimorphism in digit ratios of the left hand, when measured dorsally, differs from the evidence from earlier studies, measuring digit length ventrally, and reporting stronger sexual dimorphism in digit ratios of the right hand (1, 2, 15–17, 20, 29). A further line of studies has reported that the stronger sexual dimorphism in the 2D:4D ratio of the right hand only occurs for ventral measurements (1, 2, 29), whereas not for digit ratios derived from bone length (10, 12, 13).

The occurrence of similar patterns of sex differences in digit ratios, as measured dorsally, among children and adults supports the early determination of these sex differences. Studies have reported that the sexual dimorphic effect in the relative length of digits (2D:4D) might be related to prenatal sex-hormone levels (1, 2). However, the exact mechanism of this effect is not yet clear. Some theorists have further suggested that the ratio of prenatal androgens (testosterone) to estrogens (estradiol), i.e., the T:E ratio, may determine sexual dimorphism in digit ratios (30).

Based on a rodent model, Zheng and Cohn (31) suggested that sex differences in 2D:4D are determined by the opposing effects of androgen receptor and estrogen receptor-α activity in bone progenitor tissues of digit 4. Polymorphism in the estrogen receptor-α gene accounted for some variance (11%) in digit ratios of birds (32), although there is evidence that the epidermis only has androgen receptors, but not estrogen receptors-α (33). Moreover, testosterone injection experiments in primates during early gestational age have evidenced male-typical changes in dermatoglyphic variables (34) and in ventral, but not bone, 2D:4D ratios (35). It is therefore possible that prenatal testosterone has effects on both the bones and the epidermis, whereas estrogen effects are limited to the bones. Hence, genetic effects of estrogen receptors, together with prenatal testosterone levels, could determine sex differences in bone digit ratios, whereas the relative placing of flexion creases could depend on the latter factor, but not on the former one.

Studies have shown that the formation of the finger flexion creases likely is determined genetically (36). Because a geometric continuity (from anterior to posterior) is visible in the placing of flexion creases in a hand, the placing of flexion creases in a digit seems to be the part of a hand-wide developmental plan. Although a sexual dimorphism in the placing of flexion creases (ratio of ventral digit 3 length by hand length) has been reported (37), as of yet the relative placing of digit flexion creases itself has not been investigated. Similar to flexion creases, the relative length of fingers (i.e., digit ratios) also appears to follow a hand-wide developmental plan. To recap, the present studies show a pattern of sexual dimorphism in dorsally measured digit ratios (namely, a stronger effect in the relative length of digit 5), different from the pattern of sexual dimorphism in ventrally measured digit ratios (namely, a stronger effect in the relative length of digit 2). Accordingly, two different developmental pathways, acting within the same organ, might account for this differentiated pattern. In this context, future research would benefit from investigating both the relative placing of digit flexion creases and the relative length of digits, as measured dorsally, in order to delineate the factors determining these more clearly.

Across the current Studies I–III, sex differences in dorsally measured 2D:4D nominally were not statistically significant, although effect sizes were present (i.e., non-null) in some tests. Limited sample size (i.e., *N* < 150; similar to our samples) definitely accounts for such failures to reach conventional significance criteria in null-hypothesis significance testing, even when the size of effects is as expected. Several prior studies of comparable research also yielded nominally not significant sex effects in 2D:4D derived from bone digit length [(19, 23, 38); exception (39)], whereas larger samples usually yield findings that are statistically significant, even when effect sizes are of similar size [*N* = 327 (10); *N* = 1,060 (11); *N* = 3,172 (12); *N* = 250 (13)].

Furthermore, sex differences in the 2D:4D ratio have been reported to be larger for ventral than for bone measurements [for a meta-analysis, see Ref. (20)]. It thus appears that soft tissue, or the placing of flexion creases, or both, may be factors contributing to larger sex effects observed for ventrally measured digit ratios, as compared to bone digit ratios. It is interesting to note that some studies even reported reversed trends for bone digit ratios [i.e., higher male than female means (40)]. Seen in this perspective, the pattern of sex effects in 2D:4D in the current Studies I–III does not appear to be atypical.

In our data, the strongest sex effect among children (Study III) occurred for the 3D:5D ratio, whereas among adults (Studies I and II) for the 4D:5D ratio. This either may be due to chance (i.e., sampling error) or, alternatively, may point to additional interaction effects of age and sex on relative digit lengths. To replicate and clarify these findings remains an agenda for future research. In this context, it is informative to note that prior research has not yielded consistent patterns with regard to the magnitude of the sexual dimorphism seen in the various digit ratios (11, 14–17). Relatedly, relationships between sex-hormone levels and the 2D:4D ratio appear to be weak or absent [for a meta-analysis, see Ref. (41)]. Thus, it is likely that factors other than sex hormones impact on and confound the sexual dimorphism in digit ratios.

Dorsal digit length seems to be a suitable proxy for phalangeal bone length. A study of digit ratios derived from phalangeal bone length, including digit ratios involving digit 5, may further validate the present findings. In this context, investigations including individuals affected by endocrinologically informative conditions (such as congenital adrenal hyperplasia, complete androgen insensitivity syndrome, or Klinefelter syndrome) would be particularly interesting.

In conclusion, the evidence from the present series of studies suggests that digit ratios, when measured dorsally and involving digit 5 as a constituent of the ratio, present stronger sex differences than the widely investigated, classic, ventrally measured 2D:4D ratio.

## Author Contributions

SK and MV contributed equally to the planning of the study, analysis of the data, and intellectual input in interpretation. MS also contributed significantly in planning of the study, analysis, and interpretation of the results. All three authors are equally responsible for accuracy and originality of this work.

## Conflict of Interest Statement

The authors declare that the research was conducted in the absence of any commercial or financial relationships that could be construed as a potential conflict of interest. The reviewer SW and handling editor declared their shared affiliation, and the handling editor states that the process nevertheless met the standards of a fair and objective review.
